# Nonsurgical Extradural Hematoma in Intensive Care: Predictive Factors for Progression

**DOI:** 10.7759/cureus.87742

**Published:** 2025-07-11

**Authors:** Lamiae Bennis, Youssef Elouardi, Imane Oussayeh, Mohammed Khallouki

**Affiliations:** 1 Intensive Care Unit, Ibn Tofail Hospital, Cadi Ayyad University, Marrakesh, MAR

**Keywords:** conservative management, extradural hematoma, factors, progression, surgery

## Abstract

Introduction

Extradural hematoma (EDH) is a potentially life-threatening neurosurgical emergency that typically requires urgent surgical evacuation to prevent neurological deterioration and death. However, a subset of patients can be managed nonsurgically in the ICU with close neurological monitoring. This study aimed to identify predictive factors for EDH progression in patients managed conservatively in the ICU.

Materials and methods

We conducted a retrospective analytical study of patients admitted to the surgical ICU of Mohammed VI University Hospital Center in Marrakesh over a five-year period. The study included patients with post-traumatic EDH who were initially managed nonsurgically.

Results

A total of 61 cases were included, of which 12 patients eventually required delayed surgery. EDH progression was significantly associated with temporal location (p = 0.046), large hematoma volume (p = 0.002), coagulopathy (p = 0.04), a Glasgow Coma Scale score ≥13 at admission (p = 0.04), and early CT scanning (p = 0.0005).

Conclusions

Our findings suggest that the presence of certain predictive factors may indicate a higher risk of EDH progression. These cases require close monitoring and preparedness for surgical intervention to optimize patient outcomes.

## Introduction

Extradural hematoma (EDH), also referred to as epidural hematoma, is a neurosurgical emergency characterized by the accumulation of blood between the dura mater and the inner table of the skull, most commonly resulting from arterial injury following trauma [1]. Although EDH accounts for only 1-4% of traumatic brain injuries (TBIs) [2], it poses a significant risk of morbidity and mortality if not promptly identified and treated [3]. The classic clinical presentation includes a lucid interval followed by rapid neurological decline [4], although symptoms may vary widely depending on the hematoma’s size, location, and the presence of associated intracranial injuries [5].

Traditionally, surgical evacuation has been the standard of care, especially in cases exhibiting significant mass effect, midline shift, or neurological deficits [6]. However, recent evidence indicates that a carefully selected subset of patients with small, asymptomatic, or minimally symptomatic EDH may be safely managed nonsurgically with close neurological monitoring in the ICU [7]. This conservative strategy is typically reserved for cases where the hematoma volume is below a critical threshold (commonly <30 mL) [8], there is no substantial midline shift (<5 mm) [9], and the patient remains neurologically stable [10].

While nonsurgical management offers potential advantages - such as avoidance of surgical risks, shorter hospital stays, and reduced healthcare costs - it also carries the inherent danger of hematoma progression, which may result in delayed neurological deterioration and poorer outcomes [11]. Therefore, identifying patients at higher risk of EDH progression is essential for optimizing treatment decisions [12]. Various factors have been proposed as potential predictors of progression, including initial hematoma volume, coagulation status, the presence of skull fractures, and patient age [13-16], although current evidence remains inconsistent [17]. Furthermore, the optimal monitoring protocol - specifically, the frequency of neurological assessments and repeat imaging - has not yet been clearly defined [18].

This study aims to assess the clinical and radiological predictors of EDH progression in patients managed conservatively in the ICU. By analyzing a cohort of nonsurgically treated EDH cases, we seek to identify high-risk features that may necessitate closer monitoring or earlier surgical intervention. A better understanding of these predictors could support more informed clinical decision-making, improve patient selection for conservative management, and ultimately enhance outcomes in this vulnerable population. The findings may also contribute to the development of evidence-based guidelines for the ICU monitoring and management of EDH.

## Materials and methods

We conducted a retrospective, single-center analytical cohort study of patients with post-traumatic EDH who were initially managed conservatively in the surgical ICU of Mohamed VI University Hospital Center in Marrakesh, Morocco. The study covered a five-year period, from January 2019 to December 2023, to ensure an adequate sample size and sufficient follow-up duration.

We included patients aged ≥16 years with traumatic EDH confirmed by initial non-contrast cranial CT scan who were initially assigned to nonsurgical management under ICU monitoring. Inclusion required complete clinical and radiological data, defined by the availability of serial CT scans (at admission, six to 24 hours, and 48 hours) and complete neurological assessment records, including Glasgow Coma Scale (GCS) scores and pupillary responses. Exclusion criteria included patients aged ≤15 years, pregnant women, those who underwent immediate surgery (defined as surgical evacuation within six hours of admission), spontaneous (nontraumatic) EDH, and cases with incomplete data.

Eligible patients were identified through the ICU registry. Medical records of all eligible cases (n = 89) were reviewed, with 28 patients excluded - 22 due to immediate surgical intervention and six due to incomplete data, yielding a final cohort of 61 patients for analysis. Patients were subsequently stratified into two groups based on their clinical evolution. The Delayed Surgical Group (DG; n = 12) included patients who exhibited either radiological progression (defined as an increase of ≥2 mm in hematoma thickness on serial CT scans) or neurological deterioration (defined as a ≥2-point decrease in GCS score), necessitating surgical evacuation after initial conservative management. The Nonsurgical Management Group (NG; n = 49) included patients who maintained radiological stability (<2 mm variation in hematoma size) and neurological stability (GCS fluctuation ≤1 point) throughout the ICU monitoring period.

The study was conducted at Mohamed VI University Hospital Center, a tertiary trauma center serving southern Morocco, with continuous 24/7 neurosurgical coverage. The ICU protocol included standardized monitoring, such as hourly GCS assessments and repeat CT scans at six, 24, and 48 hours post-admission. Data were collected using a predefined operational form that captured epidemiological variables (age, sex, and mechanism of injury), clinical and paraclinical data (GCS score, neurological symptoms, hemodynamic and respiratory status, imaging findings, and hematoma volume and location), therapeutic interventions (medical treatments and extracranial surgeries), and outcome measures (hematoma progression, need for delayed surgery, complications, and mortality).

Statistical analysis was performed using Jamovi (version 2.6.26), an open-source statistical software platform. Descriptive statistics for continuous variables were reported as mean ± SD, while categorical variables were summarized using frequency distributions and percentages. Comparative analyses between the DG and NG groups were conducted using appropriate statistical tests based on variable type. The chi-square (χ²) test was used for categorical variables; Fisher’s exact test was applied when expected cell counts were below 5. Continuous variables were compared using independent samples t-tests for normally distributed data and the Mann-Whitney U test for non-normally distributed variables. A p-value of <0.05 was considered statistically significant. This multi-test strategy ensured robust and appropriate analysis across varying data types and distributions.

The study was approved by the institutional ethics committee in accordance with ethical standards for medical research.

## Results

Our study included 61 cases of post-traumatic EDH. The average patient age was 38.3 years, with a clear male predominance. Road traffic accidents were the most common mechanism of injury, accounting for 54 cases (88%). The classic clinical presentation - initial loss of consciousness followed by secondary neurological deterioration - was observed in only 18 patients (30%). Management strategies included neurosedation combined with strict control of secondary systemic brain injury. Neuromonitoring was implemented using transcranial Doppler and serial follow-up cranial CT imaging.

Based on the progression of EDH, patients were divided into two groups: the DG, which included 12 patients (20%) who required delayed surgical intervention, and the NG, which included 49 patients (80%) who remained stable under conservative management. Comparative analysis between the two groups revealed significant differences in both clinical and radiological parameters. The DG group had a significantly shorter time to the first CT scan (1.92 ± 1.06 hours vs. 5.8 ± 7.13 hours; p = 0.00058), a higher prevalence of temporal EDH localization (seven patients, 58% vs. 13 patients, 27%; p = 0.046), and a larger hematoma volume (10.65 ± 2.59 mm vs. 6.93 ± 2.58 mm; p = 0.0016). Coagulopathy was also more frequent in the DG group (four patients, 33% vs. four patients, 8%; p = 0.0409).

Interestingly, a higher proportion of DG patients presented with mild head trauma (GCS ≥13) compared to the NG group (five patients, 42% vs. five patients, 10%; p = 0.015), whereas severe trauma (GCS ≤8) was more common in the NG group (31 patients, 63% vs. four patients, 33%). These findings suggest that certain features - namely, temporal hematoma location, larger hematoma volume, presence of coagulopathy, and early imaging - may serve as predictors of EDH progression, even in patients who initially present with stable neurological status (Table 1, Table 2, Table 3, Table 4).

**Table 1:** Injury characteristics DG, Delayed Surgical Group; NG, Nonsurgical Management Group

Variable	DG (%)	NG (%)	Statistical test	t test	χ²
Side					
Left	58%	45%	p = 0.532	-	0.39
Right	42%	51%	-	-	-
Bilateral	0%	4%	-	-	-
Localization					
Frontal	25%	41%	-	-	-
Occipital	0%	10%	-	-	-
Temporal	58%	27%	p = 0.046*	-	3.716
Parietal	17%	22%	-	-	-
Volume (mean ± SD), mm	10.65 ± 2.99	6.93 ± 2.58	p = 0.0016*	3.42	-

**Table 2:** Demographic and clinical characteristics DG, Delayed Surgical Group; NG, Nonsurgical Management Group

Variable	DG	NG	Statistical test	t test	χ²
Age (mean ± SD, years)	67 ± 13.23	59 ± 18.73	p = 0.307	1.03	-
Antithrombotic therapy (%)	0%	2%	p = 1	-	NaN
Time to first CT scan (mean ± SD, hours)	1.92 ± 1.06	5.8 ± 7.13	p = 0.00058*	5.24	-

**Table 3:** Neurological and physiological parameters DG, Delayed Surgical Group; GCS, Glasgow Coma Scale; NG, Nonsurgical Management Group

Variable	DG (%)	NG (%)	Statistical test	t test	χ²
GCS					
GCS ≥13	42%	10%	p = 0.0190*	-	4.288
GCS 9-12	25%	27%	-	-	-
GCS ≤8	33%	63%	-	-	-
SpO₂ < 90%	17%	24%	p = 0.1	-	0.31
MAP < 80-90 mmHg	8%	24%	p = 0.432	-	1.22

**Table 4:** Secondary injuries and outcomes DG, Delayed Surgical Group; NG, Nonsurgical Management Group

Variable	DG (%)	NG (%)	Statistical test	t test	χ²
Extracranial injuries	25%	51%	p = 0.122	-	2.37
Depressed skull fracture	8%	17%	p = 0.432	-	0.892
Subarachnoid hemorrhage	58%	61%	p = 1	-	0
Brain contusion	75%	71%	p = 1	-	0
Subdural hematoma	42%	29%	p = 0.489	-	0.48
Cerebral edema	0%	4%	p = 1	-	0
Anemia (Hb < 9 g/dL)	33%	14%	p = 0.202	-	1.63
Coagulopathy	33%	8%	p = 0.0409*	-	4.21
Dysnatremia	17%	18%	p = 1	-	0
Hypoxia	17%	24%	p = 0.71	-	0.14
Hypocapnia	17%	4%	p = 0.170	-	1.31
Hypercapnia	50%	29%	p = 0.182	-	1.78
Extracranial surgery	17%	18%	p = 1	-	NaN
Mortality	20%	36%	p = 0.3	-	1.07

Notably, there were no significant differences between the groups in terms of age, use of antithrombotic therapy, presence of extracranial injuries, subdural hematoma, or rates of extracranial surgery (p > 0.05 for all). Although the DG group exhibited higher rates of anemia (4 (33%) vs. 7 (14%), p = 0.202) and hypercapnia (6 (50%) vs. 14 (29%), p = 0.182), these differences were not statistically significant. Likewise, other parameters such as hypoxia, hypocapnia, and the presence of depressed skull fractures showed no significant variation between the two groups.

## Discussion

This discussion compares the various factors associated with EDH progression identified in our study with those reported in the literature, focusing on demographic characteristics, coagulopathy, neurological status, radiological features, secondary insults, associated lesions, and extracranial injuries.

Demographics presentation

Our analysis showed no statistically significant differences in age or sex between the DG and the NG, suggesting these factors do not independently influence the need for surgical intervention. The mean age of our cohort was 38.8 years, with a male predominance - findings consistent with previous studies reporting a higher prevalence of EDH in young males due to increased trauma exposure [1]. Similar age and sex distributions were observed in other conservative management studies, such as Zwayed and Lucke-Wold [7], where 62 EDH cases had a mean age of 32 years, and 74% were male. However, Rizvi et al. [19], who studied a younger cohort (mean age: 20.4 years), proposed that age may be a contributing factor to EDH progression, suggesting that younger patients could be more susceptible to deterioration.

Coagulopathy and neurological status

Coagulopathy was significantly associated with EDH progression in our study, observed in 33% of DG patients compared to 8% in NG. This aligns with findings from Mayr et al., who identified coagulopathy as an independent risk factor for hematoma expansion. The link is biologically plausible, as coagulation disorders increase the likelihood of continued bleeding, necessitating vigilant monitoring and possibly earlier surgical intervention [10]. Similarly, Talving et al. [20] reported trauma-induced coagulopathy as an independent predictor of hematoma growth.

Interestingly, mild head trauma (GCS ≥13) was more common in DG (42%), while severe trauma (GCS ≤8) predominated in NG (63%). This paradox may be explained by the delayed deterioration of initially “mild” cases due to EDH progression, a trend also reported by Basamh et al. [8], where 26% of conservatively managed EDH patients eventually required delayed surgery. In contrast, some severely injured patients may have had extensive brain damage or systemic instability that precluded surgical intervention.

Radiological predictive factors

The time to the first CT scan was significantly shorter in the DG group (1.92 ± 1.06 hours) compared to the NG group (5.8 ± 7.13 hours), consistent with findings from Rizvi et al. [19]. This suggests that the DG group either presented with more severe symptoms, prompting earlier imaging, or that institutional protocols favored rapid evaluation for high-risk presentations. Conversely, patients in the NG group may have presented with milder symptoms, resulting in delayed imaging and conservative management.

Temporal EDH localization was more frequent in DG (58%), a finding supported by anatomical considerations - temporal hematomas, located near the middle meningeal artery, carry a higher risk of progression [7]. Moreover, the average hematoma thickness was significantly greater in the DG group (10.65 ± 2.99 mm vs. 7.05 ± 2.7 mm in NG), reinforcing the notion that larger hematomas are more likely to require surgical evacuation. According to Bullock et al. [1], surgical intervention is indicated for EDH volumes >30 mL or thickness >15 mm. Similarly, Rizvi et al. [19] identified temporal EDH as a high-risk entity (p < 0.001) due to arterial vulnerability.

Secondary insults: hypoxia and metabolic derangements

Although hypoxia (SpO₂ <90%) was more common in NG (24% vs. 0%), this difference was not statistically significant. This contrasts with Chesnut et al.’s landmark study [21], which identified hypoxia as an independent predictor of mortality in TBI. Likewise, hypocapnia and hypercapnia showed no significant associations with EDH progression in our cohort, suggesting that progression may be less influenced by PaCO₂ fluctuations than in other types of brain injury, such as diffuse axonal injury.

Associated lesions and extracranial surgery

Contrary to some previous studies that found associations between EDH progression and coexisting intracranial lesions or extracranial surgeries [9,20], our findings did not show statistically significant correlations. While literature has suggested that subdural hematomas, cerebral contusions, and polytrauma requiring emergency surgery may worsen EDH through coagulopathy or hemodynamic compromise [1,8,10], our data indicate that these factors were not significantly associated with progression in conservatively managed patients. The lack of association between extracranial injuries/surgery and EDH progression (p = 1) suggests that intrinsic hematoma characteristics - such as volume and location - may be more influential than systemic factors in driving deterioration. However, larger prospective studies are needed to confirm this hypothesis and improve risk stratification.

Mortality and conservative management feasibility

There was no statistically significant difference in mortality between DG and NG, supporting the feasibility of conservative management in carefully selected cases. Zwayed and Lucke-Wold [7] similarly reported 100% survival among 62 patients with EDH managed nonsurgically (GCS 13-15 and volume <40 mm). On the other hand, Rizvi et al. [19] reported that 30.2% of initially conservatively treated patients ultimately required surgery, highlighting the importance of close monitoring - including serial CT scans within six to 48 hours - to detect progression early.

Limitations and future directions

This study has limitations, including its retrospective design and single-center setting, which may limit generalizability. Additionally, the small sample size of the DG group (n = 12) reduces statistical power in some comparisons.

Prospective, multicenter studies are needed to validate our findings and detect more subtle associations. The use of advanced imaging techniques, such as MRI susceptibility-weighted imaging, may improve risk stratification by identifying microbleeds or early markers of progression. Future research should also evaluate the role of targeted interventions - such as timely correction of coagulopathy or optimized scheduling of extracranial surgeries - in improving EDH outcomes. Finally, there is a need for evidence-based guidelines regarding optimal monitoring intervals and surgical thresholds in polytrauma patients with EDH to help standardize care and optimize outcomes.

## Conclusions

EDH progression remains a significant concern in the management of TBI, especially in intensive care settings. Our study provides valuable insights into the predictive factors of EDH progression. By incorporating these findings into clinical practice -through strict patient selection criteria for conservative management and implementation of structured imaging protocols - clinicians can improve decision-making and potentially enhance patient outcomes.
